# Measuring the stress tensor in nitrogen-doped CVD diamond using solid-state quantum sensor

**DOI:** 10.1080/14686996.2025.2546779

**Published:** 2025-08-18

**Authors:** T. Tsuji, S. Harada, T. Teraji

**Affiliations:** a International Center for Young Scientists, National Institute for Materials Science, Tsukuba, Ibaraki, Japan; b Center for Integrated Research of Future Electronics (CIRFE), Institute of Materials and Systems for Sustainability (IMaSS), Nagoya University, Nagoya, Japan; c Department of Materials Process Engineering, Nagoya University, Nagoya, Japan; d Research Center for Electronic and Optical Materials, National Institute for Materials Science, Tsukuba, Ibaraki, Japan

**Keywords:** CVD diamond, nitrogen dope, NV center, stress

## Abstract

We measured the residual stress tensor in a nitrogen-doped chemical vapor deposition (001) diamond film. The stress tensor was evaluated from the amount of the shift in optically detected magnetic resonance (ODMR) spectra of NV center in the diamond. A confocal microscopy setup was used to observe the spatial variation of the stress tensor in the diamond film. We found that the components of the stress tensor, σ_xy_, σ_yz_, σ_zx_ and σ_xx_+ σ_yy_+ σ_zz_, of the residual stress were approximately 0.077, −0.39, −0.67 and 1.52 GPa, respectively, in the x = [100], y = [010], z = [001] coordinate system. Regarding the components of the shear stress, σ_xy_, σ_yz_ and σ_zx_, the nitrogen-doped CVD diamond film grown in this study had mainly sheared stress in the z-direction, which was the growth direction of the CVD diamond film. In addition, regarding axial stress σ_xx_+ σ_yy_+ σ_zz_, the CVD diamond film was subjected to compressive stress. Due to this compressive stress, the volume of the CVD diamond film decreased by approximately 0.073%. We considered that nitrogen doping contributed to the decrease in volume of the CVD diamond film.

## IMPACT STATEMENT

We measured the residual stress tensor in a nitrogen-doped CVD diamond film. We found that the film was subjected to shear stress in the growth direction and compressive stress.

## GRAPHICAL ABSTRACT

**Figure:**
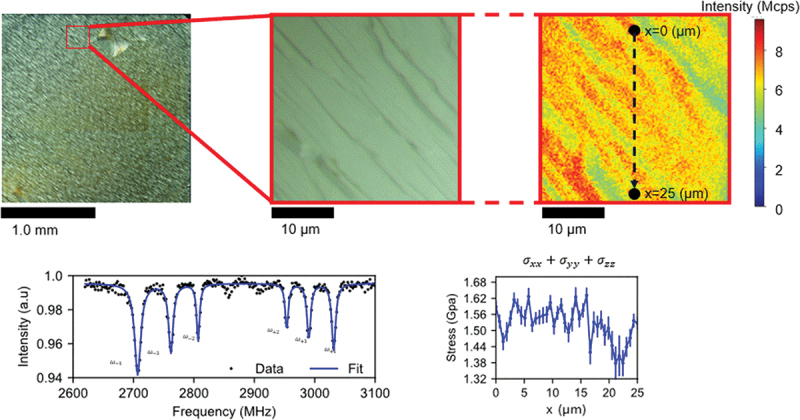

## Introduction

Chemical vapor deposition (CVD) nitrogen-doped diamond is an interesting material for quantum application because nitrogen-vacancy (NV) centers [1] are promising color centers for quantum sensors [2,3], networks [4–6] and computers [7,8]. Regarding the quantum sensors, NV centers are sensitive to a wide range of physical parameters, such as the magnetic field [9,10], electric field [11,12], temperature [13,14] and pressure [15–17].

The CVD diamond films are typically subject to residual stress [18,19]. However, such stress affects the performance of quantum applications. For example, the stress alters the resonance frequency of the NV centers [20,21], which reportedly deteriorates the properties of quantum network and computers [22]. In addition, inhomogeneous resonance frequencies of NV centers lead to a decrease of the spin dephasing time *T*_2_^*^ of the NV centers [23], which deteriorates the sensitivity of quantum sensors [24]. Therefore, reducing the residual stress in nitrogen-doped diamond films is fundamentally important for the realization of quantum applications. For this purpose, evaluation of residual stress is essential.

**Figure 1.**
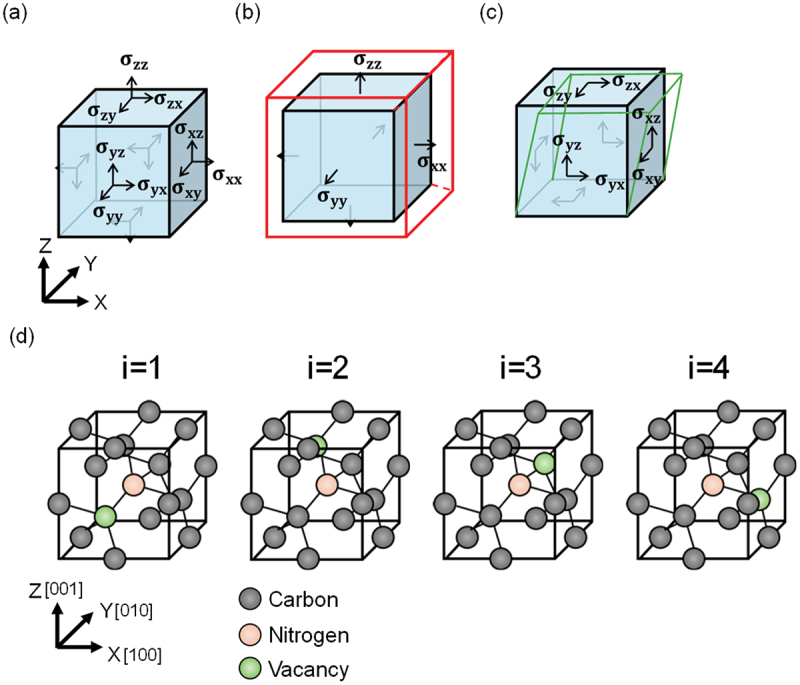
(a) Schematic of the unit cell of the crystal and stress. (b) Schematic of the unit cell of the crystal under axial stress components (σ_xx_, σ_yy_, σ_zz_): a crystal subjected to axial stress, transitioning from a blue to a red cell. (c) Schematic of the unit cell of the crystal under shear stress components (σ_xy_, σ_yz_, σ_zx_): a crystal subjected to shear stress undergoes, transitioning from a blue to a green cell. (d) NV centers in the diamond with four different axes.

Stress in a crystal is applied along the x-axis, y-axis and z-axis, corresponding to the x-plane, y-plane and z-plane in a crystal, respectively, as shown in Figure 1(a). Thus, the stress in a crystal is represented as the following matrix, known as the stress tensor.

$$\left[\begin{array}{ccc} \sigma_{\mathrm{xx}} & \sigma_{\mathrm{xy}} & \sigma_{\mathrm{xz}}\\ \sigma_{\mathrm{xy}} & \sigma_{\mathrm{yy}} & \sigma_{\mathrm{yz}}\\ \sigma_{\mathrm{xz}} & \sigma_{\mathrm{yz}} & \sigma_{\mathrm{zz}} \end{array}\right]$$

The stress tensor in a crystal is a 3 × 3 symmetric matrix, which contains six independent components (σ_xx_, σ_yy_, σ_zz,_ σ_xy_, σ_yz_, σ_zx_). As shown in Figure 1(b), the sum of diagonal components (σ_xx_+ σ_yy_+σ_zz_) represents axial stresses, corresponding to the volumetric change in the crystal as described later. The non-diagonal components (σ_xy_, σ_yz_, σ_zx_), known as shear stresses, describe changes in the crystal’s shape, as shown in Figure 1(c).

Although it is important to evaluate the components of the stress tensor, it has been challenging to measure them. Conventional methods for measuring stress have been represented by Raman spectroscopy [25], birefringence microscopy [26], X-ray diffraction [27] and electron backscatter diffraction (EBSD) [28]. Raman spectroscopy evaluates stress by measuring Raman peak shifts caused by changes in lattice vibrations. The analysis generally assumes only hydrostatic pressure is present ($\sigma_{\mathrm{xx}}=\sigma_{\mathrm{yy}}=\sigma_{\mathrm{zz}},\;\sigma_{\mathrm{xy}}=\sigma_{\mathrm{yz}}=\sigma_{\mathrm{xz}}=0$). Using confocal optics, Raman spectroscopy can detect the stress for three-dimensional mapping [25]. Birefringence microscopy senses stress through optical detection of refractive index changes caused by strain using transmitted light. Birefringence microscopy is sensitive to the in-plane shear component of the stress tensor, integrated across the sample thickness [26]. X-ray topography (XRT) detects strain as contrast variations in X-ray diffraction images, highlighting areas of large local strain or defects. X-ray topography does not detect individual components of the stress tensor [27]. EBSD detects stress by analyzing distortions in Kikuchi patterns using a scanning electron microscope. EBSD is sensitive to shear stress components near the surface of the materials due to the penetration depth of the electron beam [28].

In contrast, this study enabled the direct extraction of the components (σ_xy_, σ_yz_, σ_zx_, σ_xx_+σ_yy_+σ_zz_) of the stress tensor without any assumption by using a color center in diamonds, nitrogen-vacancy (NV) center. The measurement principle has been reported [29–31] as described below. NV center is a point defect in diamond with a spin-1 electronic ground state. When stress is applied to the NV center, the two resonance frequencies of a single NV center shift, which is referred to as the spin-mechanical interaction between the NV center’s electron spin and the stress tensor. Both nitrogen and vacancies are in carbon substitution positions, and when adjacent, they constitute NV centers. The direction of the NV center is, therefore, one of the four bonding hand directions of the diamond crystal (corresponding to i = 1,2,3,4 in Figure 1(d)). Thus, the resonance frequencies changed by a given stress tensor are 4 (directions) × 2 = 8 in total. By reading these eight resonance frequencies by optically detected magnetic resonance (ODMR) scheme, which is the way to detect resonance frequencies optically by applying microwaves to the NV centers [32], components of the stress tensor (σ_xy_, σ_yz_, σ_zx_, σ_xx_+σ_yy_+σ_zz_) can be uniquely constructed [29,31].

In this study, we applied this stress-measuring technique using NV centers to evaluate the stress tensor (σ_xy_, σ_yz_, σ_zx_, σ_xx_+σ_yy_+σ_zz_) of the residual stress in the nitrogen-doped CVD diamond film with confocal microscopy. We selected the area in the CVD film with low defect density to measure the residual stress in the CVD film, which was evaluated by using XRT image. In addition, we discuss the volume change of CVD diamond due to this stress and whether nitrogen atoms in the CVD diamond were one of the underlying causes of this residual stress.

## Method

A high-pressure and high-temperature (HPHT) type-Ib (100) single crystal with a thickness of 500 μm was used as a substrate. The top surface of the substrate was mechanically polished along [110] direction under fine polishing conditions. The CVD growth conditions are as follows: 110 Torr reaction pressure, 1.4 kW microwave power, 10% ^12^C purified methane concentration ratio (flow rate ratio of CH_4_ to the total gas flow), 10% nitrogen concentration ratio (flow rate ratio of N_2_ to the total gas flow), 2% oxygen concentration (flow rate ratio of O_2_ to the total gas flow), and 1020–1090°C substrate temperature. In this study, oxygen adding growth condition was applied because oxygen adding has a role of decreasing the number of newly generated dislocations in the CVD thin film [31–35]. A 20 μm-thick homoepitaxial diamond was obtained.

**Figure 2.**
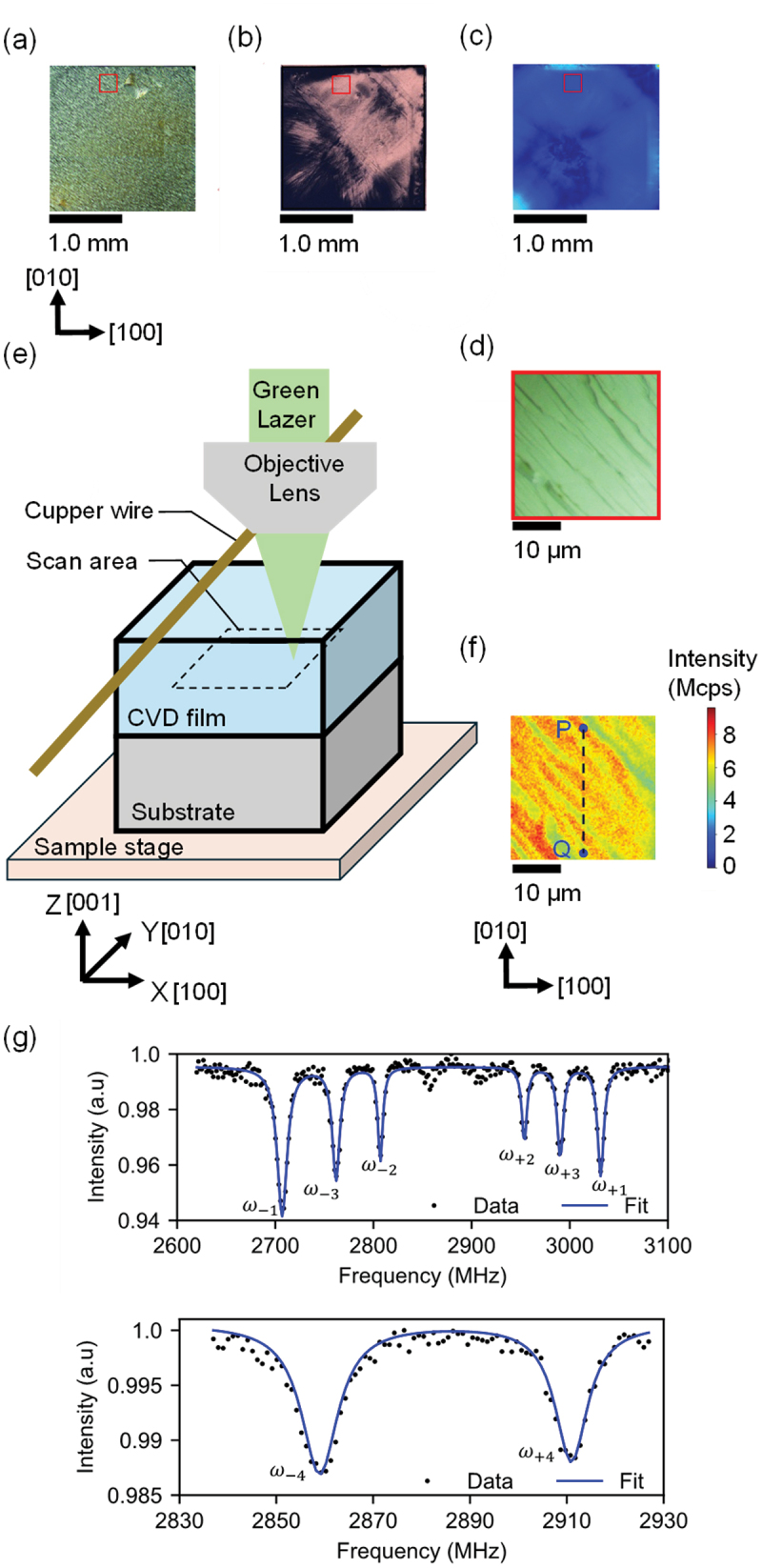
(a)–(c) Optical microscope image, X-ray topography image and birefringence image of the CVD diamond film, respectively. (d) Optical microscope image of the CVD diamond film in red-squared area shown in Figure 1(a). (e) Schematic of a confocal microscope for measuring the residual stress tensor. By moving the sample stage with the diamond, the fluorescence detection point was moved. (f) The fluorescence image of the XY plane within the CVD diamond film. (g) ODMR spectrum measured at the positions P shown in Figure 2(f).

Grazing incident reflection synchrotron XRT was performed at the BL8S2 beamline of the Aichi Synchrotron Radiation Center, Japan, using a monochromatic X-ray beam of 14.3 keV. The applied diffraction vector (g vector) was **g** = $\left[\overset{ˉ}{2}\overset{ˉ}{2}4\right]$. Topographic images were recorded on nuclear emulsion plates and digitally captured using an optical microscope with transmission illumination. Figure 2(a–c) shows an optical microscope image, X-ray topography image and birefringence image of the whole area of CVD diamond film, respectively. We intentionally selected the area with low defect density, marked by the red squares (260 μm × 260 μm) in Figure 2(a–c) in order to measure the residual stress in the CVD diamond film. We measured the stress tensor in this area. The step bunching feature was observed along the [110] direction, indicating lateral growth was predominant in the CVD growth as shown in Figure 2(d).

The fluorescence from the NV center was detected using a confocal microscope. The configuration of the diamond sample, objective lens (Evident, PLAPON60XO) with numerical aperture of 1.42, and copper wire during measurement are shown in Figure 2(e). The diamond sample was mounted on a sample stage that could be scanned in the x, y and z directions. Figure 2(f) shows the fluorescence images in the XY plane of CVD diamond film. This image was measured at a depth of 10 μm from the surface of the CVD film. Here, the surface of the CVD diamond film and the interface between the CVD film diamond and the substrate may affect the NV center properties. Thus, we performed measurements of the stress tensor at the center of the CVD diamond thin-film surface and thin-film/substrate interface. The position of 10 μm was deep from the surface of the CVD diamond film of 20 μm, where these effects were the most minimal. Moreover, the depth resolution of this confocal measurement system was approximately 1 μm, enabling accurate stress evaluation at a depth of 10 μm. A spin coherence time *T*_*2*_ of the NV center is inversely proportional to the nitrogen density $\left[N\right]$ in the diamond as $T_{2}=\alpha /\left[N\right]$ (α=160μ s⋅ppm) [36]. *T*_*2*_ measured by the spin echo sequence in the area of Figure 2(f) was approximately 13 μs. (Pulse sequence and spin-echo decay fringe of NV center measured in the CVD diamond film was described in the Supplement material.) Thus, nitrogen density in the CVD film was estimated to be approximately 13 ppm. The conversion ratio of the nitrogen density to NV center density in the CVD diamond film has been reported to be approximately 1% [37,38]. Thus, the density of NV center was estimated to be approximately 0.1 ppm in the area of Figure 2(f).

We briefly explain the procedure to obtain the stress tensor image experimentally. The NV center’s electronic ground state Hamiltonian in the presence of both stress and a static magnetic field is described as

(1) $$\begin{array}{c} H=\left(D+M_{z_{i}}\right)S_{z_{i}}^{2}+\gamma \vec{B}\cdot \vec{S_{i}}-M_{X_{i}}\left(S_{X_{i}}^{2}-S_{Y_{i}}^{2}\right)\text{\textbackslash{}break}\;\;\;\;\;\;\;\;\;\;\;\;\;\;\;\;\;\;\;\;\;\;\;\;\;\;\;\;\;\;\;+M_{Y_{i}}\left(S_{X_{i}}S_{Y_{i}}+S_{Y_{i}}S_{X_{i}}\right), \end{array}$$

where D= 2.87 GHz is the temperature-dependent zero-field splitting parameter, γ = 28.03 GHz/T is the NV gyromagnetic ratio, $\vec{S_{i}}=\left(S_{X_{i}},S_{Y_{i}},S_{Z_{i}}\right)$ are the spin-1 operators, $\vec{B}$ is the applied magnetic field and $\vec{M}=\left(M_{X_{i}},M_{Y_{i}},M_{Z_{i}}\right)$ is spin–stress interaction. The resonance frequencies ${\left(\omega_{\pm }\right)}_{i}\left(i=1,2,3,4\right)$ of each direction of the NV center are given by [29]

$${\left(\omega_{\pm }\right)}_{i}=D+M_{Z_{i}}\pm \sqrt{{\left(\gamma B_{\mathrm{Zi}}\right)}^{2}+{\left(M_{X_{i}}\right)}^{2}+{\left(M_{Y_{i}}\right)}^{2}}$$

where $B_{\mathrm{Zi}}\left(i=1,2,3,4\right)$ is the magnetic field applied to the parallel to each NV center axis shown in Figure 1(d). First, the degeneracy of the energy states of the NV centers in the four directions corresponding to the crystal bonding directions was removed by applying a static magnetic field. In this situation, eight resonance frequencies are observable in the continuous-wave optically detected magnetic resonance (ODMR) spectrum. We used a 514 nm Laser (Vortran, STRADUS514–60) with polarization aligned in the [010] direction. The CVD diamond film was irradiated with a laser of approximately 3 mW. Considering the NV center density of 0.1 ppm and the detection volume of 0.5 μm^3^ in the confocal microscope used in this study, it was estimated that there were approximately 10^4^ NV centers at one measurement point. A copper wire with a diameter of 20 μm was used for microwave irradiation. We used a samarium-cobalt magnet with surface flux magnetic density of 210 mT, which was cylindrical shape of Φ32 × 7 (mm) to apply magnetic field. The magnet was placed at a distance of approximately 5 cm from the CVD diamond. Magnetic field of approximately 7.2 mT was applied to the CVD diamond. The intensity of this magnetic field was measured with a tesla meter (Lakeshore, F71 Multi-Axis Teslameter). Figure 2(g) shows the ODMR spectra measured at the positions P of the diamond sample, as shown in Figure 2(f). The eight resonance frequencies $\left(\omega_{\pm i}\right)\left(i=1,2,3,4\right)$ were determined by fitting the ODMR spectrum with eight Lorentzian functions. Here, i denotes the number corresponding to the direction of the NV center, as shown in Figure 1(d). We defined the average frequency $S_{i}$ as

$$\begin{array}{c} S_{i}=\frac{\omega_{+i}+\omega_{-i}}{2}. \end{array}$$

We determined the three shear stress components (σ_xy_, σ_yz_, σ_zx_) and the sum of the axial stress components (σ_xx +_ σ_yy +_ σ_zz_) in the x = [100], y = [010], z = [001] coordinate system using the following equations [29]

$$\sigma_{\mathrm{xy}}=\left(S_{1}-S_{2}-S_{3}+S_{4}\right)/8a_{2}$$

$$\sigma_{\mathrm{yz}}=\left(S_{1}+S_{2}-S_{3}-S_{4}\right)/8a_{2}$$

$$\sigma_{\mathrm{zx}}=\left(S_{1}-S_{2}+S_{3}-S_{4}\right)/8a_{2}$$

$$\sigma_{\mathrm{xx}}+\sigma_{\mathrm{yy}}+\sigma_{\mathrm{zz}}=\left(\frac{S_{1}+S_{2}+S_{3}+S_{4}}{4}-D\right)/a_{1}$$

where the stress susceptibility parameters are $a_{1}$ = 4.86, $a_{2}$ = −3.7 (MHz/GPa) and the temperature-dependent zero-field splitting parameter is *D* = 2780 (MHz) (The derivation of these equations was described in the supplementary material.). Here, compressive stress and tensile stress were defined as positive and negative, respectively. Finally, by scanning the sample stage with respect to the laser irradiation position and measuring the resonance frequencies at each sample position according to the procedure described above, the components of the stress tensor σ_xy_, σ_yz_, σ_zx_ and σ_xx_ + σ_yy_ + σ_zz_ at each position were obtained.

## Result and discussion

**Figure 3.**
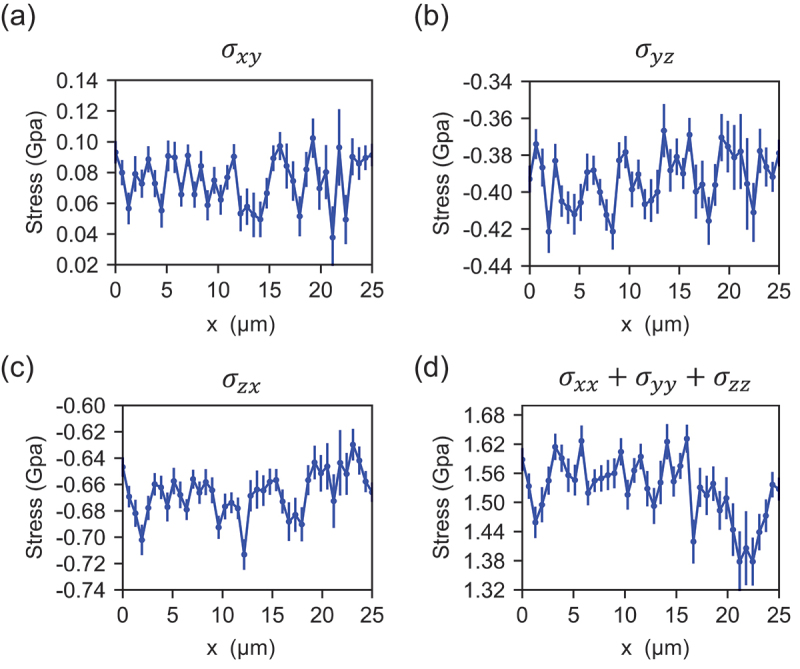
(a)–(d) The changes in the stress tensor components of σ_xy_, σ_yz_, σ_zx_ and σ_xx +_ σ_yy +_ σ_zz_ between line segment PQ, respectively. Here, x = 0 (μm) corresponds to point P and x = 25 (μm) corresponds to point Q.

We measured the stress tensor along the line between points P–Q, indicated in the fluorescence intensity mapping image shown in Figure 2(f). Figure 3 (a–d) illustrates the values of the stress tensor components of σ_xy_, σ_yz_, σ_zx_ and σ_xx +_ σ_yy +_ σ_zz_ between the line PQ, respectively. Here, x = 0 [μm] corresponds to point P and x = 25 [μm] corresponds to point Q. The error bars show the measurement error obtained using the following equations,

$${S_{i}}_{\mathrm{error}}\left(i=1,2,3,4\right)=\;\sqrt{{\left(\omega_{+i_{\mathrm{err}}}\right)}^{2}+{\left(\omega_{-i_{\mathrm{err}}}\right)}^{2}}/2$$

$${\sigma_{\mathrm{xy}}}_{\mathrm{error}}={\sigma_{\mathrm{yz}}}_{\mathrm{error}}={\sigma_{\mathrm{zx}}}_{\mathrm{error}}=\sqrt{\overset{4}{\underset{i=0}{\sum }}{\left({S_{i}}_{\mathrm{error}}\right)}^{2}}/8a_{2}$$

$${\left(\sigma_{\mathrm{xx}}+\sigma_{\mathrm{yy}}+\sigma_{\mathrm{zz}}\right)}_{\mathrm{error}}=\sqrt{\frac{\sum_{i=0}^{4}{\left({S_{i}}_{\mathrm{error}}\right)}^{2}}{4}+D_{err}^{2}}/a_{1}$$

where $\omega_{+i_{\mathrm{err}}}$ and $\omega_{-i_{\mathrm{err}}}$ were the fitting error of the resonance frequencies ${\left(\omega_{\pm }\right)}_{i}$. The average (standard deviation) of σ_xy_, σ_yz_, σ_zx_ and σ_xx_+σ_yy_+ σ_zz_ between line PQ were approximately 0.077 (0.015), −0.39 (0.02), −0.67 (0.02) and 1.52 (0.05) GPa, respectively. We found that these stress values exist as residual stress within the nitrogen-doped CVD diamond film.

**Figure 4.**
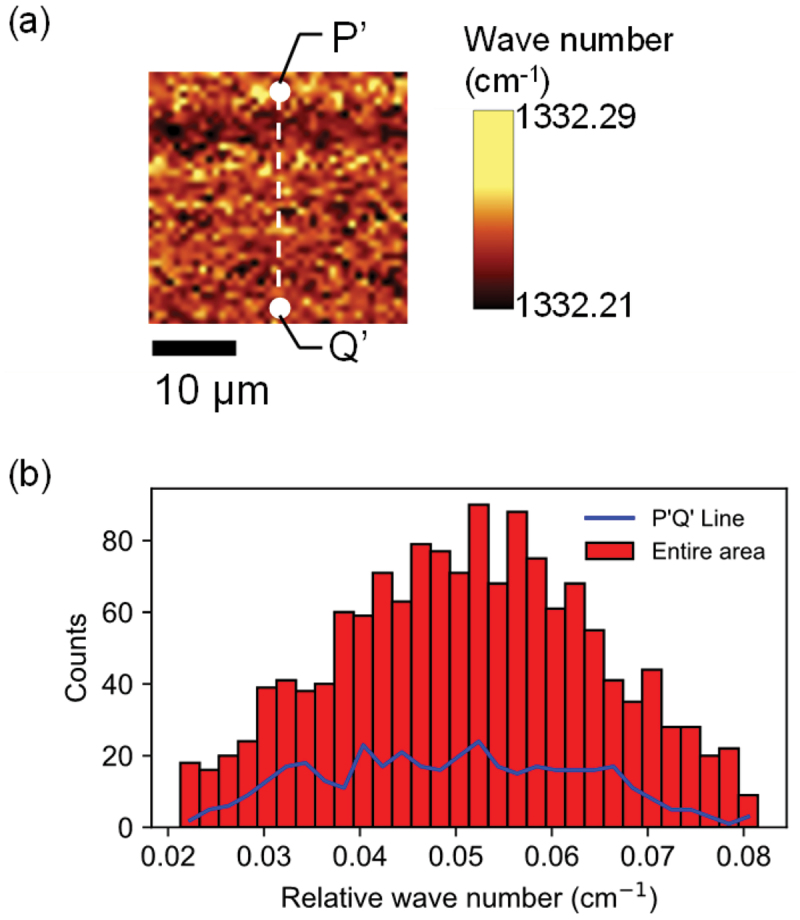
(a) Image of the shifts of Raman peak measured in the area shown in Figure 2(f). (b) Frequency distribution of Raman peak: the red box shows the frequency distribution of Raman peak shifts over the entire area measured in Figure 4(a), and the blue line shows the frequency distribution of Raman peak shifts measured on the P’Q’ line shown in Figure 4(a). Here, the wave number shown on the horizontal axis is the relative wave number to 1332.22 cm^−1^.

In order to verify whether the stress tensor measured between line PQ represented the residual stress tensor in the entire area shown in Figure 2(f), we evaluated the stress in this entire area using Raman spectroscopy. Figure 4(a) shows the image of the shift of Raman peak measured in the area shown in Figure 2(f) (The experimental method was described in detail in our previous study [20].). Figure 4(b) shows the frequency distribution of Raman peak shifts measured over the entire area measured in Figure 4(a) and that measured on the P’Q’ line shown in Figure 4(a). The average value and the standard deviation of the Raman peak shifts measured over the entire area measured in Figure 4(a) were 1332.252 and 0.016 cm^−1^, respectively. The average value and standard deviation of the Raman peak shifts measured on the line P’Q’ in Figure 4(a) were 1332.250 and 0.015 cm^−1^, respectively. Thus, the distribution of stress on the line PQ was comparable to that of this entire area shown in Figure 4(a). Therefore, we consider that the average values of the components of the stress tensor in the line PQs measured by NV centers are the representative values of the residual stress in the entire area. In addition, the 260 μm × 260 μm area in the red square shown in Figure 2(b) had uniform distribution of stress due to the small variation of signal contrast. Therefore, the stress measured between PQ line in the small area (25 μm × 25 μm) shown in Figure 2(f) was considered to be a representative value of stress in the entire area (260 μm × 260 μm) shown in Figure 2(b).

We evaluated the strain in the nitrogen-doped CVD diamond film using the stress tensor. The components of the strain tensor ($\varepsilon_{\mathrm{xx}},\;\varepsilon_{\mathrm{yy}},\;\varepsilon_{\mathrm{zz}},\;\varepsilon_{\mathrm{xy}},\varepsilon_{\mathrm{yz}},\;\varepsilon_{\mathrm{zx}}$) in diamond in the x = [100], y = [010], z = [001] coordinate system are expressed as

(2) $$\begin{array}{c} \left(\begin{array}{c} \varepsilon_{\mathrm{xx}}\\ \varepsilon_{\mathrm{yy}}\\ \varepsilon_{\mathrm{zz}}\\ \varepsilon_{\mathrm{xy}}\\ \varepsilon_{\mathrm{yz}}\\ \varepsilon_{\mathrm{zx}} \end{array}\;\right)=\left(\begin{array}{cccccc} s_{11} & s_{12} & s_{12} & 0 & 0 & 0\\ s_{12} & s_{11} & s_{12} & 0 & 0 & 0\\ s_{12} & s_{12} & s_{11} & 0 & 0 & 0\\ 0 & 0 & 0 & s_{44} & 0 & 0\\ 0 & 0 & 0 & 0 & s_{44} & 0\\ 0 & 0 & 0 & 0 & 0 & s_{44} \end{array}\;\right)\left(\begin{array}{c} \sigma_{\mathrm{xx}}\\ \sigma_{\mathrm{yy}}\\ \sigma_{\mathrm{zz}}\\ \sigma_{\mathrm{xy}}\\ \sigma_{\mathrm{yz}}\\ \sigma_{\mathrm{zx}} \end{array}\;\right) \end{array}$$

where $s_{11}$ = 0.952, $s_{12}$ = −0.099 and $s_{44}$ = 1.737 (10^−3^ GPa^−1^) are elastic compliance constants of diamond [39]. Using this equation, the components of the shear strain, $\varepsilon_{\mathrm{xy}}$, $\varepsilon_{\mathrm{yz}}$ and $\varepsilon_{\mathrm{zx}}$, were estimated to be 0.013%, 0.067% and 0.12%, respectively. Thus, $\varepsilon_{\mathrm{yz}}$ and $\varepsilon_{\mathrm{zx}}$ exhibited strains that were, respectively, six times and ten times greater than $\varepsilon_{\mathrm{xy}}$. This means that, regarding shear strain, the nitrogen-doped CVD diamond film grown in this study had mainly sheared strain in the z-direction, which was the growth direction of the CVD diamond film.

Next, we estimated the volumetric change of the CVD diamond film by components of the stress tensor obtained in this study using Equation (2). The volumetric change ε_v_ is defined as the fractional change in volume:

$$\epsilon_{v}=\frac{\;\mathrm{\Delta V}}{V}=\frac{V{\;}^{\prime }-V}{V}$$

where V is the initial volume, and V′ is the final volume through deformation due to stress applied.

The initial volume is described using a small volume element dx,dy,dz for each direction

V=dx⋅dy⋅dz.

A small volume element after being deformed by stress is described as follows,

$$dx^{\prime }=\;\left(1\;+\;\varepsilon_{\mathrm{xx}}\right)\mathrm{dx},dy^{\prime }=\;\left(1\;+\;\varepsilon_{\mathrm{yy}}\right)\mathrm{dy},dz^{\prime }=\;\left(1\;+\;\varepsilon_{\mathrm{zz}}\right)\mathrm{dz}.$$

thus, the final volume is

$$V{\;}^{\prime }=dx^{\prime }\cdot dy^{\prime }\cdot dz^{\prime }=\;\left(1\;+\;\varepsilon_{\mathrm{xx}}\right)\left(1\;+\;\varepsilon_{\mathrm{yy}}\right)\left(1\;+\;\varepsilon_{\mathrm{zz}}\right)\mathrm{dx}\cdot \mathrm{dy}\cdot \mathrm{dz}\;.$$

For small deformations, higher-order terms can be neglected, so:

$$V{\;}^{\prime }\approx V\left(1\;+\;\varepsilon_{\mathrm{xx}}+\;\varepsilon_{\mathrm{yy}}+\;\varepsilon_{\mathrm{zz}}\right)\;.$$

The volumetric change is then:

(3) $$\begin{array}{c} \epsilon_{v}=\frac{V{\;}^{\prime }-V}{V}=\varepsilon_{\mathrm{xx}}+\varepsilon_{\mathrm{yy}}+\varepsilon_{\mathrm{zz}}\;. \end{array}$$

Thus, the volumetric change is expressed as following using Equation (2)

$$\epsilon_{v}=\;\varepsilon_{\mathrm{xx}}+\;\varepsilon_{\mathrm{yy}}+\;\varepsilon_{\mathrm{zz}}=\left(s_{11}+2s_{12}\right)\left(\sigma_{\mathrm{xx}}+\;\sigma_{\mathrm{yy}}+\;\sigma_{\mathrm{zz}}\right).$$

Thus, the volume of the CVD diamond film was estimated to decrease by approximately $\varepsilon_{v}$ = 0.073% due to the compressive stress.

Here, we discuss whether this volume change was due to the incorporation of nitrogen into the CVD film by comparing the lattice constants of CVD diamond film with different doping atoms. A previous study [40] has reported the lattice constants of four diamond samples: (001) Ib HPHT diamond (sample A), ^12^C purified undoped CVD diamond grown on the (001) Ib HPHT diamond (sample B), boron-doped CVD diamond grown on the (001) Ib HPHT diamond (sample C) and phosphorus-doped CVD diamond grown on the (001) Ib HPHT diamond (sample D). The lattice constants of samples A, B, C, and D were 3.567115, 3.567135, 3.570113 and 3.567128 Å, respectively. The strain of these CVD films $\varepsilon_{\mathrm{cvd}}$ with respect to the (001) Ib HPHT diamond is calculated as

$$\varepsilon_{\mathrm{cvd}}=1-\frac{l_{\mathrm{CVD}}}{l_{\mathrm{HPHT}}}$$

where $l_{\mathrm{CVD}}$ and $l_{\mathrm{HPHT}}$ are lattice constant of CVD diamond and (100) Ib HPHT diamond, respectively. $\varepsilon_{\mathrm{cvd}}$ of samples B, C, D were estimated to approximately −5.6 × 10^−6^, −8.4 × 10^−4^, −3.6 × 10^−6^, respectively. These results indicated that samples B, C and D were under tensile stress. On the other hand, as mentioned earlier, the nitrogen-doped CVD diamond film measured in this study was under compressive stress of approximately 1.52 GPa. Therefore, we considered that nitrogen doping in CVD diamond film contributed to the compressive stress which caused the decrease in volume of the CVD diamond film. Our previous study also reported that (111) nitrogen-doped CVD diamond film on the (111) Ib HPHT diamond were subject to compressive stress [20]. In addition, there is another study reported that nitrogen has an atomic radius less than that of carbon atoms and it preferentially forms a lone-pair with one of its four carbon atoms, resulting in the distortion of the surrounding diamond lattice [41]. These reports are consistent with our results.

We consider that the stress tensor measured in this study was caused by the difference in nitrogen density [N] between the diamond substrate, including [N] of approximately 100 ppm and the CVD diamond film including [N] of approximately 13 ppm. Thus, reducing this difference in nitrogen density is considered important for reduction of the residual stress. Thus, a simple solution would be to use a diamond substrate whose nitrogen density is the same as that of the CVD diamond film. On the other hand, Ib (100) HPHT diamond substrates, which contain about 100 ppm nitrogen, have been typically used as diamond substrates for CVD diamond films [42,43]. However, if the nitrogen density of the CVD film is 100 ppm, the same as that of the Ib substrate, the spin coherence time *T*_*2*_ (or spin dephasing time *T*_2_^*^) of the NV center becomes small, less than about 2 μs (or 100 ns) [36], which is too low to function as a quantum application [24]. Therefore, it may be effective to gradually decrease the nitrogen density in the CVD film from the interface between the substrate and the CVD film to the surface of the CVD film to reduce the residual stress for sensor applications.

## Conclusion

We evaluated the residual stress tensor in a nitrogen-doped chemical vapor deposition (001) diamond film using NV center. We found that the components of the stress tensor, σ_xy_, σ_yz_, σ_zx_ and σ_xx_+σ_yy_+σ_zz_, of the residual stress were approximately 0.077, −0.39, −0.67 and 1.52 GPa, respectively, in the x = [100], y = [010], z = [001] coordinate system. From these values, the shear strains, $\varepsilon_{\mathrm{xy}}$, $\varepsilon_{\mathrm{yz}}$ and $\varepsilon_{\mathrm{zx}}$ were estimated to be 0.013%, 0.067% and 0.12%, respectively. This means that, regarding shear strain, the nitrogen-doped CVD diamond film grown in this study had mainly sheared strain in the z-direction, which was the growth direction of the CVD diamond film. In addition, the volume of the CVD diamond film decreased by 0.073% due to the compressive stress of σ_xx_+ σ_yy_+ σ_zz_. We considered that nitrogen doping contributed to the decrease in volume of the CVD diamond film. We believe that these stress tensor evaluations will pave the way for effective reduction of stress in the diamond.

## Supplementary Material

Supplemental Material
