# Supplementary material for: Measuring the stress tensor in nitrogen-doped CVD diamond using solid-state quantum sensor
Source: Sci Technol Adv Mater. 2025 Aug 18;26(1):2546779. doi: 10.1080/14686996.2025.2546779 (PMC12409865; doi:10.1080/14686996.2025.2546779)
Supplement: Supplemental Material [file TSTA_A_2546779_SM4982.docx]

**Supplemental Materials for “Measuring the stress tensor in nitrogen-doped CVD diamond using solid-state quantum sensor”**

T. Tsuji^1^, S. Harada^2^, T. Teraji^1*^

1. *National Institute for Materials Science, 1-1 Namiki, Tsukuba, Ibaraki 305-0044, Japan*
2. *Nagoya University, Furo-cho, Nagoya 464-8601, Japan* TERAJI.Tokuyuki@nims.go.jp

# A. Spin-echo measurement

A spin coherence time *T_2_* measured by spin-echo sequence has been inversely proportional to nitrogen density [𝑁] in the diamond as 𝑇
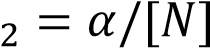
 (
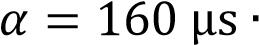
 ppm)

[36]. Figs.S1 (a) and (b) show the spin-echo pulse sequence used in this study and spinecho decay fringe of NV center measured in the CVD diamond film, respectively. The

τ p

decay envelope was fitted to the C_0_exp [− (_𝑇_2) ] to extract *T_2_. T_2_* measured by spin echo sequence in this CVD film was 12.6 ± 0.9 μs. Thus, nitrogen density in the CVD film was estimated to be approximately 12.7 ± 1.9 ppm.


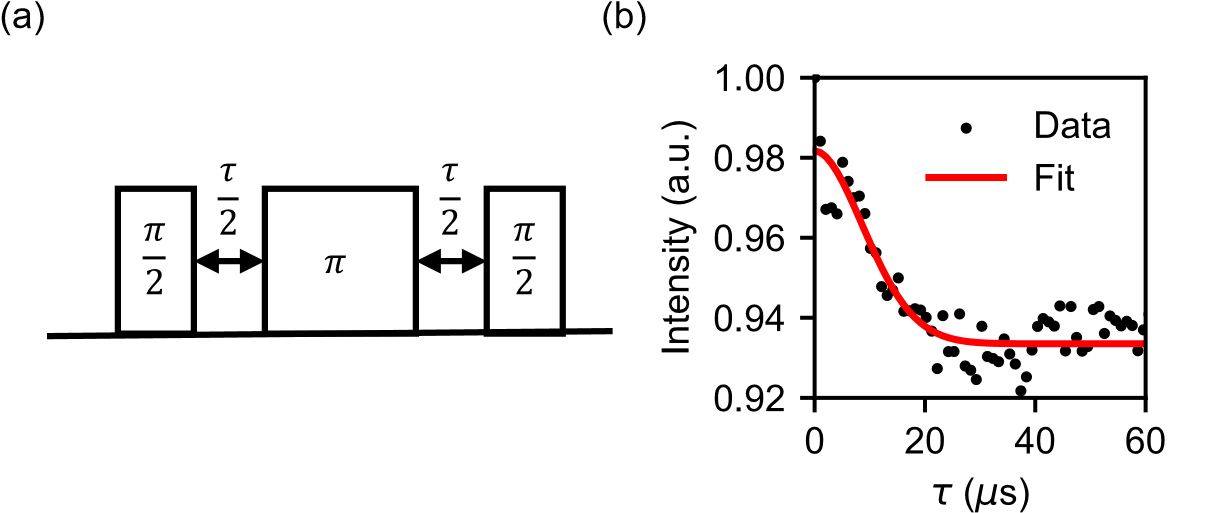


Figure.S1 (a)-(d)the spin-echo pulse sequence used in this study and spin-echo decay fringe of NV center measured in the CVD diamond film, respectively.

B. The derivation of equations (5)-(8) in the main text.

The resonance frequencies (𝜔_±_)_𝑖_ (𝑖 = 1,2,3,4) of each direction of NV center are given by


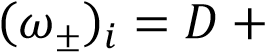
 𝑀_𝑍𝑖_ (𝛾𝐵_𝑍𝑖_)

±

√

2

+

(

𝑀

𝑋

𝑖

)

2

+

(

𝑀

𝑌

𝑖

)

2

where 𝐷 =2.87GHz is the temperature-dependent zero-field splitting parameter, 𝛾=28.03 GHz/T is the NV gyromagnetic ratio, ⃗𝑆⃗⃗ _𝑖_
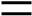
 (𝑆_𝑋𝑖_, 𝑆_𝑌𝑖_, 𝑆_𝑍𝑖_) are the spin-1 operators, 𝑀⃗⃗ = (𝑀_𝑋𝑖_, 𝑀_𝑌𝑖_, 𝑀_𝑍𝑖_) is spin-stress interaction, 𝐵_𝑍𝑖_ (𝑖 = 1,2,3,4) is the magnetic field applied to the parallel to each NV center axis shown in Fig.1 (d). We defined the average frequency 𝑆_𝑖_ as

𝜔
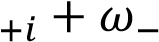
_𝑖_

𝑆_𝑖_ = = 𝐷 + 𝑀_𝑍𝑖_.


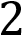


The relation between 𝑀_𝑍𝑖_ and the components of stress tensor 𝜎_𝑥𝑥_, 𝜎_𝑦𝑦_, 𝜎_𝑧𝑧_, 𝜎_𝑥𝑦_, 𝜎_𝑦𝑧_, 𝜎_𝑧𝑥_ in the x=[100], y=[010], z=[001] coordinate system are described as [1,2]

𝑀𝑧1 = 𝑎1(𝜎𝑥𝑥 + 𝜎𝑦𝑦 + 𝜎𝑧𝑧) + 2𝑎2(𝜎𝑥𝑦 + 𝜎𝑥𝑧 + 𝜎𝑦𝑧)

𝑀𝑧2 = 𝑎1(𝜎𝑥𝑥 + 𝜎𝑦𝑦 + 𝜎𝑧𝑧) + 2𝑎2(−𝜎𝑥𝑦 − 𝜎𝑥𝑧 + 𝜎𝑦𝑧)

𝑀𝑧3 = 𝑎1(𝜎𝑥𝑥 + 𝜎𝑦𝑦 + 𝜎𝑧𝑧) + 2𝑎2(−𝜎𝑥𝑦 + 𝜎𝑥𝑧 − 𝜎𝑦𝑧)

𝑀𝑧4 = 𝑎1(𝜎𝑥𝑥 + 𝜎𝑦𝑦 + 𝜎𝑧𝑧) + 2𝑎2(𝜎𝑥𝑦 − 𝜎𝑥𝑧 − 𝜎𝑦𝑧)

where the stress susceptibility parameters are 𝑎_1_= 4.86, 𝑎_2_= -3.7 (MHz/GPa). Thus, the three shear stress components (σ_xy_, σ_yz_, σ_zx_) and the sum of axial stress components (σ_xx +_ σ_yy +_ σ_zz_) shown in main text are described as

𝜎_𝑥𝑦_ = (𝑆_1_ − 𝑆_2_ − 𝑆_3_ + 𝑆_4_)/8𝑎_2_

𝜎_𝑦𝑧_ = (𝑆_1_ + 𝑆_2_ − 𝑆_3_ − 𝑆_4_)/8𝑎_2_

𝜎_𝑧𝑥_ = (𝑆_1_ − 𝑆_2_ + 𝑆_3_ − 𝑆_4_)/8𝑎_2_

𝑆
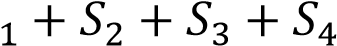


𝜎_𝑥𝑥_ + 𝜎_𝑦𝑦_ + 𝜎_𝑧𝑧_ = ( − 𝐷)/𝑎_1_.


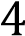


# Reference

1. D.A. Broadway, B.C. Johnson, M.S.J. Barson, S.E. Lillie, N. Dontschuk, D.J. McCloskey, A. Tsai, T. Teraji, D.A. Simpson, A. Stacey, J.C. McCallum, J.E. Bradby, M.W. Doherty, L.C.L. Hollenberg, J.-P. Tetienne, Microscopic Imaging of the Stress Tensor in Diamond Using in Situ Quantum Sensors, Nano Lett. 19 (2019) 4543–4550. https://doi.org/10.1021/acs.nanolett.9b01402.
2. P. Kehayias, M.J. Turner, R. Trubko, J.M. Schloss, C.A. Hart, M. Wesson, D.R. Glenn, R.L. Walsworth, Imaging crystal stress in diamond using ensembles of nitrogen-vacancy centers, Phys. Rev. B Condens. Matter 100 (2019) 174103.

https://doi.org/10.1103/PhysRevB.100.174103.
